# Supplementary material for: Low affinity uniporter carrier proteins can increase net substrate uptake rate by reducing efflux
Source: Sci Rep. 2018 Apr 3;8:5576. doi: 10.1038/s41598-018-23528-7 (PMC5883050; doi:10.1038/s41598-018-23528-7)
Supplement: Supplementary file 1 — Supplementary Information [file 41598_2018_23528_MOESM1_ESM.pdf]

Supplementary Information to: Low affinity uniporter carrier proteins can increase net substrate uptake rate by reducing efflux

Evert Bosdriesz[1,3,+], Meike T Wortel[1,4,+], Jurgen R Haanstra[1], Marijke J Wagner[1], Pilar de la Torre Cortés[2], and Bas Teusink[1,*]

[1]Systems Bioinformatics, Amsterdam Institute for Molecules, Medicines and Systems (AIMMS), VU University, Amsterdam, De Boelelaan 1108, 1081HZ, The Netherlands
[2]Department of Biotechnology, Delft University of Technology, Delft, The Netherlands
[3]Present address: Division of Molecular Carcinogenesis, The Netherlands Cancer Institute, Plesmanlaan 121, 1066 CX Amsterdam, The Netherlands
[4]Present address: Centre for Ecological and Evolutionary Synthesis (CEES), the Department of Biosciences, University of Oslo, Blindernveien 31, 0371 Oslo, Norway
[*]b.teusink@vu.nl
[+]These authors contributed equally

Supplementary Text

Symmetric transport model

Derivation of the rate equation of symmetric transport in terms of first order rate constants

The transporter can be in any of four states, the binding-site facing outward, with and without substrate bound, es_e and e_e, respectively, and inward facing with and without substrate bound, es_i and e_i. Assuming that binding and unbinding is much faster than the movement of the binding site over the membrane, we can use the quasi-steady state approximation for the fraction of carriers that have substrate bound to them, both inside and outside,

$$es_x = \frac{\frac{s_x}{K_D}}{1+\frac{s_x}{K_D}} e_{x,tot} \tag{S1}$$

$$e_x = \frac{1}{1+\frac{s_x}{K_D}} e_{x,tot}, \tag{S2}$$

where K_D is the substrate-transporter dissociation constant and $e_{x,tot} = es_x + e_x$ is the total number of transporters with their binding site facing the x site of the membrane (i.e. $x = e$ or $x = i$).

By definition, in steady state $e_{x,tot}$ is constant. This gives rise to the equality $k_2 es_e + k_4 e_e = k_2 es_i + k_4 e_i$. Defining

$$\sigma_x \equiv \frac{k_2 \frac{s_x}{K_D} + k_4}{1+\frac{s_x}{K_D}} \tag{S3}$$

and solving the steady state condition gives an expression for the total amount of outward and inward facing carriers, normalized to the total amount of transporters:

$$e_{e,tot} = \frac{\sigma_i}{\sigma_e + \sigma_i} e_{tot}, \tag{S4}$$

$$e_{i,tot} = \frac{\sigma_e}{\sigma_e + \sigma_i} e_{tot} \tag{S5}$$

The net uptake rate is than given by:

$$\begin{aligned} v &= k_2 \left[es_e - es_i\right] \\ &= k_2 \left[\frac{\frac{s_e}{K_D}}{1+\frac{s_e}{K_D}} \sigma_i - \frac{\frac{s_i}{K_D}}{1+\frac{s_i}{K_D}} \sigma_e\right] \frac{1}{\sigma_e + \sigma_i} e_{tot} \\ &= k_2 \left[\frac{s_e}{K_D}\left(1+\frac{s_i}{K_D}\right)\sigma_i - \frac{s_i}{K_D}\left(1+\frac{s_e}{K_D}\right)\sigma_e\right] \frac{1}{\left(1+\frac{s_i}{K_D}\right)\left(1+\frac{s_e}{K_D}\right)(\sigma_e+\sigma_i)} e_{tot} \end{aligned}$$

Filling in the σs, the term within the square brackets reduces to

$$\frac{k_4}{K_D}(s_e - s_i)$$

and the denominator to

$$2k_4 + (k_2+k_4)\frac{s_e}{K_D} + (k_2+k_4)\frac{s_i}{K_D} + 2k_2 \frac{s_e \cdot s_i}{K_D^2}.$$

Hence, the rate equation in terms of the first order rate constants is given by

$$v = e_{tot} \frac{\frac{k_2}{2K_D}(s_e - s_i)}{1 + \frac{k_2+k_4}{2k_4 K_D} s_e + \frac{k_2+k_4}{2k_4 K_D} s_i + \frac{k_2}{k_4}\frac{s_e \cdot s_i}{K_D^2}} \tag{S6}$$

Defining the macroscopic kinetic parameters

$$k_{cat} \equiv \frac{k_2 k_4}{k_2 + k_4} \tag{S7a}$$

$$K_M \equiv \frac{2k_4 K_D}{k_2 + k_4} \tag{S7b}$$

$$\alpha \equiv \frac{4k_2 k_4}{(k_2+k_4)^2} \tag{S7c}$$

than gives the rate equation

$$v = e_{tot} \frac{\frac{k_{cat}}{K_M}(s_e - s_i)}{1 + \frac{s_e}{K_M} + \frac{s_i}{K_M} + \alpha \frac{s_e \cdot s_i}{K_M^2}} \tag{S8}$$

Derivation of the optimal affinity, K_M^{opt}

In order to find the optimal K_M, K_M^{opt}, we simply take the derivative of v with respect to K_M and set that to zero. Since we have

$$\frac{\mathrm{d}v}{\mathrm{d}K_M} = -e_{tot} k_{cat} \frac{\frac{1}{K_M^2}(s_e - s_i)\left(1 + \alpha \frac{s_e \cdot s_i}{K_M^2} - 2\alpha \frac{s_e \cdot s_i}{K_M}\right)}{\left(1 + \frac{s_e}{K_M} + \frac{s_i}{K_M} + \alpha \frac{s_e \cdot s_i}{K_M^2}\right)^2}, \tag{S9}$$

K_M^{opt} is found by solving

$$0 = 1 + \alpha \frac{s_e \cdot s_i}{K_M^2} - 2\alpha \frac{s_e \cdot s_i}{K_M}. \tag{S10}$$

The physical (*i.e.* positive) solution to this quadric equation is

$$\boxed{k_M^{opt} = \sqrt{\alpha \cdot s_e \cdot s_i}} \tag{S11}$$

In comparison, the reversible Michaelis-Menten rate equation*,

$$v_{MM} = e_t \frac{\frac{k_{cat}}{K_M}(s_e - s_i)}{1 + \frac{s_e}{K_M} + \frac{s_i}{K_M}} \tag{S12}$$

does not have an optimal affinity. Reducing the K_M will always increase the rate.

*Here, we use $K_{eq} = 1$ and assume symmetry between s_e and s_i release.

The non-symmetric carrier model

In this section we study if the conclusions in the main text hold when the assumptions underlying the simplification of the rate equation are dropped. The more general rate equation describing the net transport rate of a facilitated diffusion process takes the following form (equation IX-46 in[?]):

$$v = e_t \frac{\frac{k_{cat}}{K_{M,e}}\left(s_e - s_i\right)}{1 + \frac{s_e}{K_{M,e}} + \frac{s_i}{K_{M,i}} + \alpha \frac{s_e}{K_{M,e}} \frac{s_i}{K_{M,i}}} \tag{S13}$$

where the macroscopic kinetic parameters, k_{cat}, $K_{M,e}$, $K_{M,i}$ and α, can be expressed in terms of the first order rate constants.

$$k_{cat} = \frac{k_{1f}k_{2f}k_{3f}k_{4f}}{k_{1f}\left(k_{2f}k_{3f} + k_{2f}k_{4f} + k_{2r}k_{4f} + k_{3f}k_{4f}\right)} \tag{S14a}$$

$$K_{M,e} = \frac{\left(k_{4f} + k_{4r}\right)\left(k_{1r}k_{2r} + k_{1r}k_{3f} + k_{2f}k_{3f}\right)}{k_{1f}\left(k_{2f}k_{3f} + k_{2f}k_{4f} + k_{2r}k_{4f} + k_{3f}k_{4f}\right)} \tag{S14b}$$

$$K_{M,i} = \frac{\left(k_{4f} + k_{4r}\right)\left(k_{1r}k_{2r} + k_{1r}k_{3f} + k_{2f}k_{3f}\right)}{k_{3r}\left(k_{1r}k_{2r} + k_{1r}k_{4r} + k_{2f}k_{4r} + k_{2r}k_{4r}\right)} \tag{S14c}$$

$$\alpha = \frac{\left(k_{2f} + k_{2r}\right)\left(k_{4f} + k_{4r}\right)\left(k_{1r}k_{2r} + k_{1r}k_{3f} + k_{2f}k_{3f}\right)}{\left(k_{2f}k_{3f} + k_{2f}k_{4f} + k_{2r}k_{4f} + k_{3f}k_{4f}\right)\left(k_{1r}k_{2r} + k_{1r}k_{4r} + k_{2f}k_{4r} + k_{2r}k_{4r}\right)} \tag{S14d}$$

Furthermore, the first order rate equations are related through the equilibrium constant. Since we are considering facilitated diffusion, no free energy dissipation is coupled to the transport process,*i.e.* $K_{eq} = 1$, and we have:

$$1 = K_{eq} \equiv \frac{k_{1f}k_{2f}k_{3f}k_{4f}}{k_{1r}k_{2r}k_{3r}k_{4r}}. \tag{S15}$$

This poses a constraint on the first order rate constants. Practically, this means that a mutation that affects *e.g.* the strength of extracellular substrate to carrier binding must also affect some of the other steps in the transport cycle (*e.g.* intracellular substrate release). Combined with the complicated dependency of the macroscopic parameters on the first order rate constants, an analytical approach is unfeasible. We therefore employed a parameter sampling approach to gauge to what extent our conclusions about the rate-affinity trade-off and the substrate efflux hypothesis are valid for this more general rate equation.

The rate-affinity trade-off

As in the main text, the parameter sampling approach gives a mixed picture about the theoretical underpinnings of the rate-affinity trade-off. Figure S1 shows scatterplots of k_{cat} versus affinity (defined as $1/K_{M,e}$) for randomly sampled sets of first order rate constants, with a number of different constraints assumed for some of these. In the absence of any constraints, there is a clear negative correlation between the k_{cat} and the affinity (figure S1A). However, this is not a true Pareto-front, as it appears as though there is always a possibility that the k_{cat} is enhanced without reducing the affinity (or vice verse). The fact that not the whole $k_{cat} - 1/K_M$ space is filled is due to the finite numbers of samples rather than due to a true constraint. On the other hand, if we assume that there is a (biophysical) limitation on the rate of substrate-transporter binding (k_{1f}), (*e.g.* the diffusion limit), we do find a true Pareto front (figure S1B), the location of which depends on the actual maximal k_{1f}-value. However, this conclusion does not hold if other rate constants are assumed to have some biophysical limit, as shown by the examples of restricted k_{2f} (figure S1C) or a restricted substrate-transporter dissociation constant $K_{D,e}$ ($\equiv k_{1r}/k_{1f}$, figure S1D). All in all, there are reasonable theoretical arguments to be made for a rate-affinity trade-off, but the logic is not water-tight.

Enhanced uptake by reduced efflux

In the analysis in the main text above we made the biologically motivated, simplifying assumption that the transporter is symmetric. However, our reasoning does not critically dependent on this symmetry, since it is a general property of this scheme that the substrate and product bind to different states of the transporter. Moreover, since all first order rate are interdependent through constraint (S15), $K_{M,i}$ and $K_{M,e}$ are expected to be correlated. To test this, we randomly sampled all first order rate constants from a log-normal distribution and rescaled them such that $K_{eq} = 1$ and $k_{cat} = 1$ (for details, cf. Appendix). These parameters were used to calculate the $K_{M,e}$, $K_{M,i}$ and the net steady state uptake rate J under conditions of high an low external substrate ($s_e = 100$ and $s_e = 1$, respectively). The results are depicted in figure S2. Indeed, $K_{M,e}$ and $K_{M,i}$ are correlated, albeit not strongly (Spearman correlation = 0.64, Figure S2A). More importantly, however, there appears to be an s_e-dependent optimal affinity (figure S2B). Furthermore, the set of parameters that has the highest J under low substrate conditions, performs relatively poorly under high substrate conditions (large, light red dot), and vice verse (blue dot).

Parameter sampling procedure

Sets of parameters were constructed by drawing the first order rate constant randomly from a log-normal distributions. This was done in a way such that constraint (S15) is satisfied. These parameter sets were used to calculate the steady state uptake rate (given by equation (S13)) and macroscopic kinetic parameters (given by equation (S14)). The parameter sampling was performed in Wolfram Mathematica 9.0 using the functions RandomVariate and LogNormalDistribution, which has the probability density function (PDF):

$$\frac{\exp\left(\frac{(\ln(x)-\mu)^2}{2\sigma^2}\right)}{\sqrt{2\pi}\sigma\mu} \tag{S16}$$

Rate affinity trade-off

To generate the data depicted in figure S1, for each subfigure 10 000 parameter sets were constructed. Each parameter set was constructed in the following way:

- Two sets of four numbers, $\mathscr{X} \equiv \{x_1, x_2, x_3, x_4\}$ and $\mathscr{Y} \equiv \{y_1, y_2, y_3, y_4\}$ were randomly drawn from a log-normal distribution given by the PDF (S16) with $\mu = 0$ and $\sigma = 2$.
- For **figure S1A**, there are no restrictions on individual rate constants. The set of forward rate constants, $\mathscr{K}_f \equiv \{k_{1f}, k_{2f}, k_{3f}, k_{4f}\}$ is just given by the first set, $\mathscr{K}_f = \mathscr{X}$. To get the reverse rate constants $\mathscr{K}_r \equiv \{k_{1r}, k_{2r}, k_{3r}, k_{4r}\}$, $\mathscr{Y}$ needs by be rescaled by a factor

$$a = \prod_{i=1}^{4}\left(\frac{x_i}{y_i}\right)^{1/4}. \tag{S17}$$

  This is to ensure that $K_{eq} = 1$. Hence, $\mathscr{K}_r = a \cdot \mathscr{Y}$.

- For **figure S1B** we also need to ensure that k_{1f} is restricted to some constant value c. We set the first order rate forward constants to $\mathscr{K}_f = \frac{c}{x_1} \cdot \mathscr{X}$ and $\mathscr{K}_r = \frac{c}{x_1} \cdot a \cdot \mathscr{Y}$. Similarly, for **figure S1C** we need to restrict k_{2f} to c. We use $\mathscr{K}_f = \frac{c}{x_2} \cdot \mathscr{X}$ and $\mathscr{K}_r = \frac{c}{x_2} \cdot a \cdot \mathscr{Y}$
- For **figure S1D**, the $K_{D,e}$ is fixed to a constant value c. Here, we used for the forward rate constant simply $\mathscr{K}_f = \mathscr{X}$. Since $K_{D,e} \equiv k_{1r}/k_{1f}$, we set $k_{1r} = c \cdot x_1$. Defining $\beta = \left(\frac{x_2 x_3 x_4}{y_2 y_2 y_4}\frac{1}{c}\right)^{1/3}$ and setting $\{k_{2r}, k_{3r}, k_{4r}\} = \beta \cdot \{y_2, y_3, y_4\}$ additionally ensures $K_{eq} = 1$.

Enhanced uptake by non-symmetric low affinity transporters

Figures S2 A and B are constructed from the same 10000 parameter sets. Each set was constructed such that $k_{cat} = 1$ and $K_{eq} = 1$ as follows:

- Two sets of four numbers, $\mathscr{X} \equiv \{x_1, x_2, x_3, x_4\}$ and $\mathscr{Y} \equiv \{y_1, y_2, y_3, y_4\}$ were randomly drawn from a log-normal distribution given by the by the PDF (S16) with $\mu = 0$ and $\sigma = 2$.
- The set $\mathscr{Y}'$ is obtained by rescaling $\mathscr{Y}$ by a factor

$$a = \prod_{i=1}^{4}\left(\frac{x_i}{y_i}\right)^{1/4} \tag{S18}$$

  to obtain $\mathscr{Y}' \equiv a \cdot \mathscr{Y}$. This ensures $\prod_{i=1}^{4} x_i/y'_i = 1$.

- Both sets are rescaled by a factor $1/\tilde{k}_{cat}$, as defined in equation (S7a) with $k_{if} \to x_i$ and $k_{ir} \to y'_i$,*i.e.*

$$\tilde{k}_{cat} = \frac{x_1 x_2 x_3 x_4}{x_1\left(x_2 x_3 + x_2 x_4 + y'_2 x_4 + x_3 x_4\right)} \tag{S19}$$

  This rescaling is done to ensure that $k_{cat} = 1$ for each parameter set. The first order rate constants are thus $\mathscr{K}_f = \frac{1}{\tilde{k}_{cat}}\mathscr{X}$ and $\mathscr{K}_r = \frac{a}{\tilde{k}_{cat}}\mathscr{Y}$.

Supplementary Tables

Name	Genotype	Relevant phenotype	Origin
CEN.PK113-7D	*MATa URA3 HIS3 LEU2 TRP1 MAL2-8c SUC2*	Prototrophic control strain	Euroscarf
EBY.VW4000	MATa ura3 his3 leu2 trp1 MAL2-8c SUC2 hxt13Δ::loxP hxt15Δ::loxP hxt16Δ::loxP hxt14Δ::loxP hxt12Δ::loxP hxt9Δ::loxP hxt11Δ::loxP hxt10Δ::loxP hxt8Δ::loxP hxt514Δ::loxP hxt2Δ::loxP hxt367Δ::loxP gal2Δ stl1v::loxP agt1Δ::loxP ydl247wΔ::loxP yjr160cΔ::loxP	HXT null strain, auxotrophic for uracil, leucine, histidine and tryptophan. Does not grow on glucose as sole carbon source	Wieczorke et al. (1999)
IMX746	*MATa ura3 MAL2-8c SUC2 hxt13Δ::loxP hxt15Δ::loxP hxt16Δ::loxP hxt14Δ::loxP hxt12Δ::loxP hxt9Δ::loxP hxt11Δ::loxP hxt10Δ::loxP hxt8Δ::loxP hxt514Δ::loxP hxt2Δ::loxP hxt367Δ::loxP gal2Δ stl1v::loxP agt1Δ::loxP ydl247wΔ::loxP yjr160cΔ::loxP can1:: LEU2-HIS3-TRP1*	HXT null strain with a single auxotrophy for uracil	This study
IMI335	*MATa MAL2-8c SUC2 hxt13Δ::loxP hxt15Δ::loxP hxt16Δ::loxP hxt14Δ::loxP hxt12Δ::loxP hxt9Δ::loxP hxt11Δ::loxP hxt10Δ::loxP hxt8Δ::loxP hxt514Δ::loxP hxt2Δ::loxP hxt367Δ::loxP gal2Δ stl1v::loxP agt1Δ::loxP ydl247wΔ: :loxP yjr160cΔ::loxP can1::LEU2- HIS3-TRP1 ura3:: HXT7_T213N-URA3.*	Prototrophic HXT null strain expressing HXT7_T213N	This study
IMI337	*MATa MAL2-8c SUC2 hxt13Δ::loxP hxt15Δ::loxP hxt16Δ::loxP hxt14Δ::loxP hxt12Δ::loxP hxt9Δ::loxP hxt11Δ::loxP hxt10Δ::loxP hxt8Δ::loxP hxt514Δ::loxP hxt2Δ::loxP hxt367Δ::loxP gal2Δ stl1v::loxP agt1Δ::loxP ydl247wΔ::loxP yjr160cΔ::loxP can1::LEU2-HIS3-TRP1 ura3::HXT7_T213T-URA3.*	Prototrophic HXT null strain expressing HXT7_T213T	This study

Table S1. *Saccharomyces cerevisiae* strains used in this study

Overlapping sequences for homologous recombination

Name	Sequence 5' --> 3'	Purification
5890_TRP1 fw	AATAAAACGTCATATCTATGCTACAACATTCCAAAATTTGTCCC AAAAAGTCTTTGGTTCAGAGACCGAGTTAGGGACAG	HPLC
5891_TRP1 rv	GTGCCTATTGATGATCTGGCGGAATGTCTGCCGTGCCATAGC CATGCCTTCACATATAGTTGCCTCACTTGTCGCTTATG	HPLC
2335_HIS3 fw	ACTATATGTGAAGGCATGGCTATGGCACGGCAGACATTCCGC CAGATCATCAATAGGCACCTCTTGGCCTCCTCTAGTACACTC	HPLC
2336_HIS3 rv	GTTGAACATTCTTAGGCTGGTCGAATCATTTAGACACGGGCAT CGTCCTCTCGAAAGGTGTACGGAATACCACTTGCCACCTATC	HPLC
5892_LEU2 fw	CACCTTTCGAGAGGACGATGCCCGTGTCTAAATGATTCGACCA GCCTAAGAATGTTCAACTATCACGAGGCCCTTTCGTC	HPLC
7052_LEU2 rv	ATGACAAATTCAAAAGAAGACGCCGACATAGAGGAGAAGCAT ATGTACAATGAGCCGGTGGTTCACGTAGTGGGCCATCG	HPLC

Overlapping sequences for Gibson assembly

Name	Sequence 5' --> 3'	Purification
6290_pRS406 fw	GCTCTTATTGACCACACCTCTACCGGCATGCCACAGAATCAGGGGATAACG	HPLC
6291_pRS406 rv	GTTGTTGACGCTAACATTCAACGCTAGTATCACACCGCATAGGGTAATAAC	HPLC
6292_TDH3p fw	ATTATATCAGTTATTACCCTATGCGGTGTGATACTAGCGTTGAATGTTAGCGTCAAC	HPLC
6293_TDH3p rv	TTGCTCTGCAATAGCAGCGAATTCTGACATTTTGTTTGTTTATGTGTGTTTATTCGAAAC	HPLC
6294_HXT7 fw	GTTTCGAATAAACACACATAAACAAACAAAATGTCAGAATTCGCTGCTATTGC	HPLC
6295_HXT7 rv	TCTTTCCTGCGTTATCCCCTGATTCTGTGGCATGCCGGTAGAGGTGTGGTCAATAAG	HPLC

Diagnostic primers for homologous recombination

Name	Sequence 5' --> 3'	Purification
5894_diag CAN1 fw	TCGGGAGCAAGATTGTTGTG	Desalted
5895_diag TRP1 rv	GCCGTAATCATTGACCAGAG	Desalted
5896_diag TRP1 fw	CGGAGGTGTGGAGACAAATG	Desalted
5897_diag HIS3 rv	GGGCTTTCTGCTCTGTCATC	Desalted
5898_diag HIS3 fw	TCCCTCCACCAAAGGTGTTC	Desalted
5899_diag LEU2 rv	CTGTCGCCGAAGAAGTTAAG	Desalted
5900_diag LEU2 fw	TCGGCTGTGATTTCTTGACC	Desalted
5901_diag CAN1 rv	AGAAGAGTGGTTGCGAACAG	Desalted

Diagnostic primers for Gibson assembly

Name	Sequence 5' --> 3'	Purification
6034_diag TDH3p fw	CTTCTGCTCTCTCTGATTTGG	Desalted
1852_diag HXT7 rv	GTACAATGGCTTGTCATCGTGAG	Desalted
1693_diag URA3 fw	GCTGCTACTCATCCTAGTCC	Desalted
2613_diag TDH3p rv	GCATGTACGGGTTACAGCAG	Desalted
3795_diag ADH1t fw	ATGCAGCTCGAGGCGAATTTCTTATGATTTATGA	Desalted
3228_diag pRS406 rv	GCGGATAAAGTTGCAGGAC	Desalted

Table S2. Primers used in this study

Name	Relevant characteristics	Origin
Hxt7mnx-pVT_T213N	plasmid pVT-102-U expressing the *HXT7* _T213N isoform under the *ADH1* promoter	Kasahara et al (2011)
Hxt7mnx_pVT_T213T	plasmid pVT-102-U expressing the *HXT7* _T213T isoform under the *ADH1* promoter	Kasahara et al (2011)
pRS405	Template for *LEU2* gene amplification	ATCC, Sikorski et al. (1989)
pRS406	Template for Gibson assembly	ATCC, Sikorski et al. (1989)
pUDI091	*URA3*, pMB1, *Sc pTDH3-HXT7*_T213N-*tADH1*	This study
pUDI093	*URA3*, pMB1, *Sc pTDH3-HXT7*_T213T-*tADH1*	This study

Table S3. Plasmids used in this study

Supplementary figures

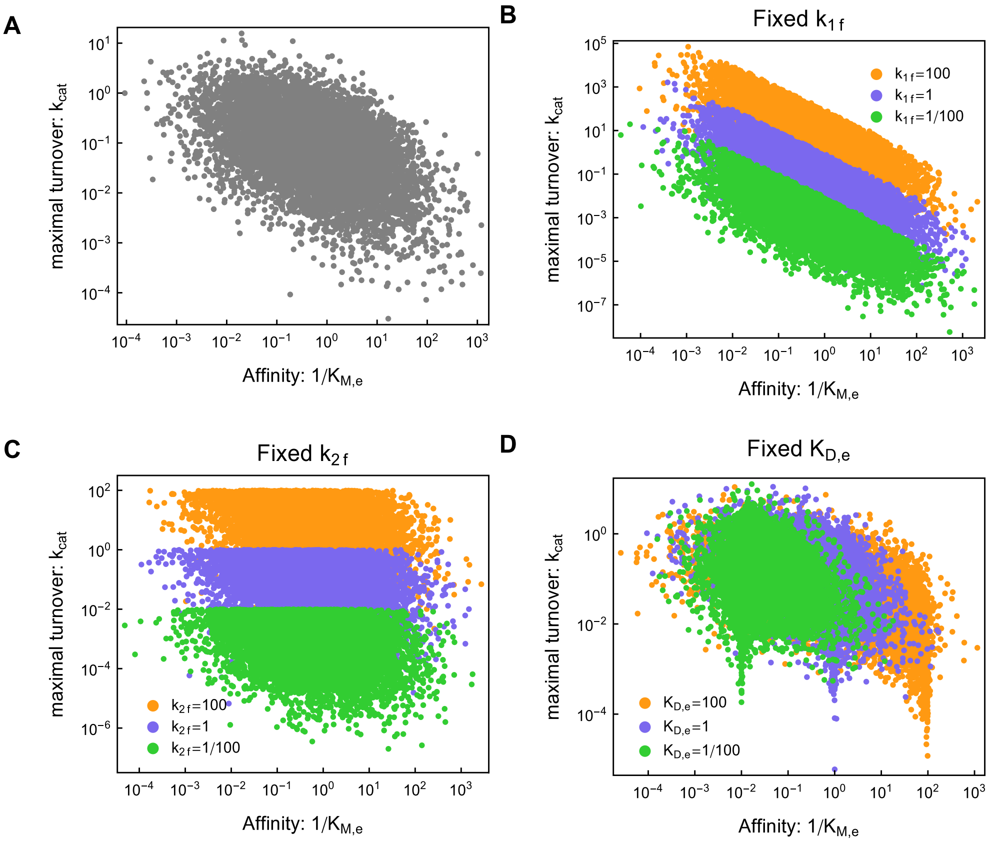

Figure S1. Parameter sampling gives inconclusive picture of potential rate-affinity tradeoff. See Supplementary Information for explanations and details on the parameter sampling procedure.

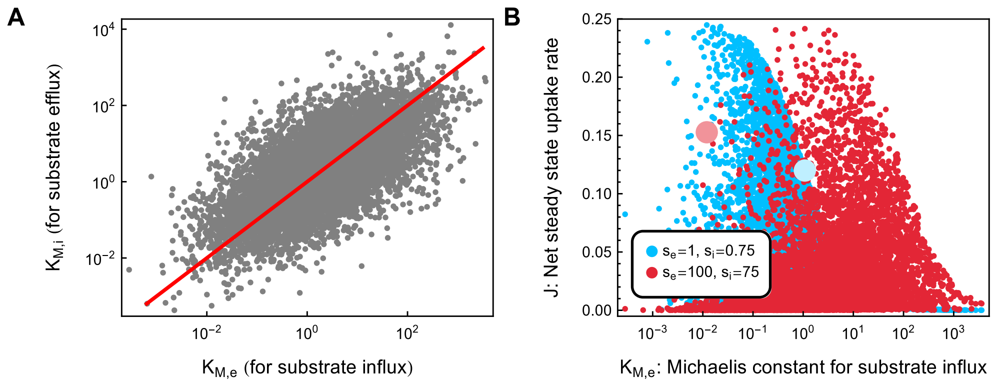

Figure S2. Parameter sampling confirms enhanced uptake by non-symmetric low affinity carriers. The large red (blue) dot in panel **B** indicate the set parameters that are optimal at low *s* (high *s*). See Supplementary Information for details on the parameter sampling procedure

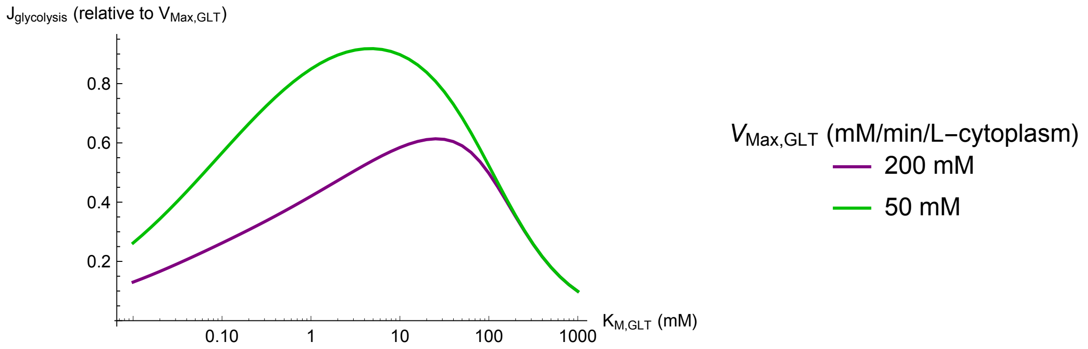

Figure S3. Effect of substrate efflux is V_{max}-dependent. Steady-state glycolytic flux relative to the transporters, $J_{glycolysis}/V_{max,GLT}$ as a function of $K_{M,GLT}$ for different values of $V_{max,GLT}$. A lower V_{max} reduces the intracellular glucose concentration. As a consequence, the effect of substrate efflux is less pronounces (the steady-state flux is closer to the V_{max}) and the optimal transporter affinity is higher. Due to this effect, the reduced-efflux hypothesis can only be tested using transporters that are comparible both in k_{cat} and V_{max}. The same model as in Fiugure 2B was used for these simulations, with [Glucose] = 110mM.

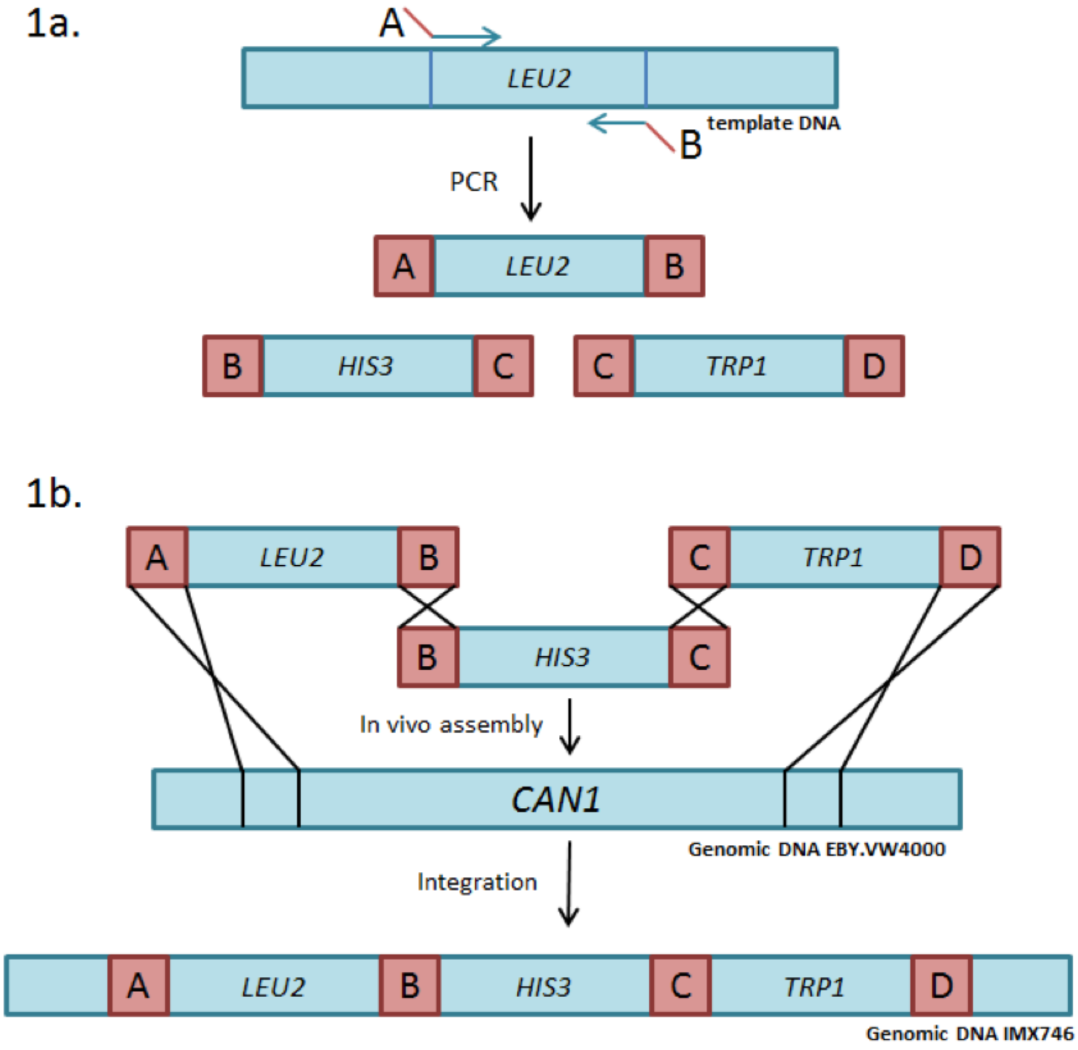

Figure S4. Repair of auxotrophies in the EBY.VW4000 strain. **A** PCR amplification of the LEU2, HIS3 and TRP1 genes with sequences overlapping either the adjacent cassettes or the integration site. **B** In vivo assembly of the marker cassettes and integration in the CAN1 locus of EBY.VW4000.
